# *StMAPK1* functions as a thermos-tolerant gene in regulating heat stress tolerance in potato (*Solanum tuberosum*)

**DOI:** 10.3389/fpls.2023.1218962

**Published:** 2023-06-20

**Authors:** Xi Zhu, Huimin Duan, Guodong Zhang, Hui Jin, Chao Xu, Shu Chen, Chuanmeng Zhou, Zhuo Chen, Jinghua Tang, Yu Zhang

**Affiliations:** ^1^ Key Laboratory of Tropical Fruit Biology, Ministry of Agriculture and Rural Affairs of China/Key Laboratory of Hainan Province for Postharvest Physiology and Technology of Tropical Horticultural Products, South Subtropical Crops Research Institute, Chinese Academy of Tropical Agricultural Sciences, Zhanjiang, China; ^2^ National Key Laboratory for Tropical Crop Breeding, Sanya Research Institute, Chinese Academy of Tropical Agricultural Sciences, Sanya, China; ^3^ Department of Biology, Xinzhou Normal University, Xinzhou, China; ^4^ Institute of Horticultural Sciences, Jiangxi Academy of Agricultural Sciences, Nanchang, China; ^5^ Grain Crop Research Institute, Yulin Academy of Agricultural Sciences, Yulin, China

**Keywords:** heat stress, potato, mitogen-activated protein kinase 1, phenotypes, photosynthesis

## Abstract

**Background and aims:**

Mitogen-activated protein kinases (MAPKs) have been reported to respond to various stimuli including heat stress. This research aimed to investigate whether *StMAPK1* is implicated in the transduction of the heat stress signal to adapt heat stress as a thermos-tolerant gene.

**Materials and methods:**

Potato plants were cultivated under mild (30°C) and acute (35°C) heat stress conditions to analyze mRNA expression of *StMAPKs* and physiological indicators. *StMAPK1* was up-regulated and down-regulated by transfection. Subcellular localization of StMAPK1 protein was observed by fluorescence microscope. The transgenic potato plants were assayed for physiological indexes, photosynthesis, cellular membrane integrity, and heat stress response gene expression.

**Results:**

Heat stress altered the expression prolife of *StMAPKs*. *StMAPK1* overexpression changed the physiological characteristics and phenotypes of potato plants under heat stresses. *StMAPK1* mediates photosynthesis and maintains membrane integrity of potato plants in response to heat stress. Stress response genes (*StP5CS*, *StCAT*, *StSOD*, and *StPOD*) in potato plants were altered by *StMAPK1* dysregulation. mRNA expression of heat stress genes (*StHSP90*, *StHSP70*, *StHSP20*, and *StHSFA3*) was affected by *StMAPK1*.

**Conclusions:**

*StMAPK1* overexpression increases the heat-tolerant capacity of potato plants at the morphological, physiological, molecular, and genetic levels.

## Highlights

1. *StMAPK1* is increasingly expressed in potato plants in response to heat stress;
2. Elevated *StMAPK1* overexpression maintains physiological characteristics and growth phenotypes of potato plants;
3. *StMAPK1* is involved in protecting photosynthesis and membrane integrity against heat stresses.

## Introduction

Climate change and global warming confer adverse effects on the crop growth and sustainable food development worldwide (Chen et al., 2020; Zandalinas et al., 2021). High temperature is one of the most predominant uncontrollable factors affecting potato yield (Handayani et al., 2019; Lee et al., 2020). Of particular, extreme heat negatively affects plant growth and crop yields, posing a significant threat to sustainable crop production. Continued greenhouse gas emissions will lead to further temperature increases, which lead to increased evaporation. This negative impact on crop yields may be aggravated in future. As consequence, the development of heat-stressed potato plants via genetic transformation is necessary to counter the negative impact of high temperature. In this scenario, identification of thermos-tolerant genes and understanding of functional mechanisms may facilitate the breeding of thermos-tolerant cultivars.

Potato (*Solanum tuberosum* L.) is the critical agricultural crop, being the third most significant crop after rice (*Oryza sativa*) and wheat (*Triticum* spp.). Additionally, high temperature negatively affects carbon synthesis (Wolf et al., 1991), photosynthesis (Wolf et al., 1990), biomass accumulation (Yubi et al., 2019), and sucrose translocation to tubers (Arreguin-Lozano and Bonner, 1949). High temperature delays the process of tuber formation and bulking (Menzel, 1983), contributes to tuber deformities and necrosis of the cultivated potato crop (Rykaczewska, 2015), and ultimately increases the risk in yield losses or quality decrease (Kim et al., 2017). In response to heat stress, plants have evolved to exhibit different strategies at the molecular, physiological, and morphological levels. Heat stress predisposes potato to oxidative stress, however, heat-tolerant potato plants display elevated antioxidant enzyme activity and altered secondary metabolism in response to heat stress (Almeselmani et al., 2006; Fogelman et al., 2019). It is still not fully understood about the mechanism by which potato plants perceive the initial heat stress and promptly respond to the high temperature at the molecular levels. The elucidation of such molecular mechanism may provide novel insights into the recognition of specific traits of potato plants responding to extreme hot climates.

Eukaryotic mitogen-activated protein kinase (MAPK or MPK) cascades play roles in relaying environmental and developmental signals into intracellular responses. Mitogen-activated protein kinases (MAPKs or MPKs) included in MAPK cascades are active in developmental processes and act in response to biotic and abiotic stresses. MAPKs are of major importance for transducing the heat stress signal to adapt to elevated temperatures (Link et al., 2002; Suri and Dhindsa, 2008). Genome sequence has identified 22 genes possibly encoding MAPKs in potato plants (Zaynab et al., 2021; Majeed et al., 2023). However, we have previously identified 15 *MAPK* genes in potato plant after aligning sequences from NCBI (Zhu et al., 2021a). The multiple sequence alignment of the conserved amino acid motif TxY divided *MAPKs* into 5 groups (Zaynab et al., 2021; Majeed et al., 2023). It has been confirmed that *MAPK1* belonging to Group B plays a positive role in response to heat stress in *Arabidopsis thaliana* (Wu et al., 2015). However, there is little knowledge of information about the molecular biological function of *MAPK1* in potato plants in response to heat stress.

Up to now, limited knowledge of heat tolerance mechanism hampered the efforts of enhancing heat stress tolerance of potatoes either by conventional or new approaches. Better understanding of key genes and overall network of genes with a role in potato thermotolerance is needed. Mitogen-activated protein kinases have been reported to respond to various stimuli including heat stress (Lin et al., 2021; Majeed et al., 2022). In this study, we aimed to investigate whether *StMAPK1* is implicated in the transduction of the heat stress signal to adapt heat stress as a thermos-tolerant gene.

## Materials and methods

### Plant growth and heat stress treatment

To analyze whether the expression profile of *StMAPKs* genes was affected by heat stress, Potato (*Solanum tuberosum* L.) cultivar “Atlantic” was cultivated under mild (30°C) and acute (35°C) heat stress conditions. Potato plants were *in vitro* planted in Murashige and Skoog (MS) medium (pH ranging among 5.8-6.0) in supplement with 3% sucrose for seedling growth or 8% sucrose for tuber generation. Potato plants were cultivated in a biotron at 22°C/15°C (day/night) with a 16-h photoperiod (2,800 Lux) and an air humidity of 50% (Zhanjiang, Guangzhou, China). Potato tubers with a sprouted bud of 1 mm in height were transferred into soil and vermiculite (1:1, v/v) pots measuring 26 cm × 27 cm × 18 cm and cultured for 5 weeks. The soil moisture remained at 70%-75%.

Heat stress is often defined as the rise in temperature beyond a threshold level for a period of time sufficient to cause irreversible damage to plant growth and development. In general, a transient elevation in temperature, usually 10-15°C above ambient, is considered heat stress. Hence, we chose 30°C and 35°C to study the response of potato plants to heat stress. The plants were cultivated under mild (30°C) and acute (35°C) heat stress conditions for 0 h, 1 h, 3 h, 6 h, 12 h, 24 h and 48 h, and the leaves were collected for detecting mRNA expression and physiological indicators. Plant height, fresh weight, dry weight, root fresh weight, and root dry weight were recorded 5 weeks after heat stress treatment.

For the quantitation of *StMAPKs* expression, there were 126 seedlings (1 line × 2 heat treatments × 7 different time periods × 3 replications × 3 pots); For the quantitation of heat-responsive genes, there were 630 seedlings (7 lines × 2 heat treatments × 5 different time periods × 3 replications × 3 pots); For the examination of physiological and photosynthetic indexes, there were 630 seedlings (7 lines × 2 heat treatments × 5 different time periods × 3 replications × 3 pots);

### Plasmid construction and transformation

To develop *StMAPK1*-transgenic potato plants (OE for short), StMAPK1 protein-encoding region (GeneBank ID: XM_015312004.1) was amplified and ligated into pBI121-EGFP plasmid using the following primers: forward, 5’-CTCGAGATGGAGGCAAGTTTAGGTGATC-3’ and reverse 5’- GTCGACGTGAGTTGGATCTGGATTAAAG-3’, provided by Bioeditas (Shaanxi, China) according to a previous method (Li et al., 2020). The constructed plasmid was introduced into *Agrobacterium tumefaciens* strains LBA4404, followed by infecting the potato tuber according to Si’s method (Si et al., 2003). In short, 12-20 week-old micro-tubers were cut into 1-2 mm discs, and submerged in 20 mL of bacterial solution for 10 min. Then the discs were transferred into shoot regeneration medium, sealed with parafilm and incubated for 2 days in dark at 26°C. *StMAPK1*-transgenic potato plants were selected using 75 mg/L of kanamycin. The transgenic potato plants were identified by sequencing PCR amplified products.

Potato plants with *StMAPK1* knocked down (RNAi for short) were constructed with a previous method (Lu et al., 2019). The sense cDNA sequence was amplified using forward primer (Kpn I) and reverse primer (EcoR I) and inserted as an Kpn I-EcoR I fragment into pHANNIBAL (pHAN-*StMAPK1*-F); The anti-sense cDNA sequence was amplified using forward primer (Hind III) and reverse primer (BamH I) and inserted as a Hind III-BamH I fragment into pHANNIBAL (pHAN-*StMAPK1*-RF). The pHAN-*StMAPK1*-RF were subcloned at Sac I and Spe I sites into pART vector (pART-*StMAPK1*-RNAi). pART-*StMAPK1*-RNAi was introduced into LBA4404 and incubated for 48 h at 28°C, followed by PCR confirmation using the specific primers for RNAi (forward: 5’-AATTCGTTCCAACCAACAATTG-3’ and reverse: 5’-AGTTGCAACCAACTGACCAGATAT-3’) and NPT II (forward: 5’-ATGACTGGGCACAACAGACAATCG-3’ and reverse: 5’-TCAGAAGAACTCGTCAAGAAGGCG-3’). The stably transgenic plants were stored in MS medium.

### Subcellular localization of StMAPK1

Protein-coding sequence of *StMAPK1* was amplified and ligated to the expression vector pCAM35s-GFP, using the following primers: forward, 5’-ATGGAGGCAAGTTTAGGTGATC-3’ and reverse, 5’-GTGAGTTGGATCTGGATTAAAG-3’. The constructed plasmid was transformed into Agrobacterium tumefaciens Gv31. The transformed strain infiltrated tobacco epidermal cells in accordance with a previous method reported by Sparkes et al. (Sparkes et al., 2006). The green fluorescence was detected 48 h after infiltration under a Leica TCA confocal scanning laser microscopy (Leica, Weztlar, Germany).

### Gene expression assay

Total RNA was extracted using TRIzol RNA Extraction Kit (Invitrogen, Carlsbad, CA, USA), following the manufacturer’s description. The first-strand cDNA was generated using the First-Strand cDNA Synthesis Kit according to the instructions from the TransGen Biotech (Beijing, China). qPCR was carried out in a reaction system consisting of 100 ng of cDNA, 10 μL of SYBR Premix Ex Taq (2 ×) (Takara, Tokyo, Japan) and 0.8 μL of specific primers (0.5 μM) on ABI3000 system (Applied Biosystems, Foster City, CA, USA). The reaction procedures were as follows, one cycle at 94°C for 2 min and 40 cycles of amplification at 94°C for 30 s, 60°C for 34 s, and 72°C for 30s. The specific primers were listed in **Table 1**. The relative mRNA expression was calculated using the formula 2^-△△Ct^. Solanum tuberosum translation elongation factor 1α gene (StEf1α) served as an internal control.

**Table 1:** List of specific primers for qRT-PCR.

Genbank accession	Gene	Forward (5’-3’)	Reverse (5’-3’)	Product length (bp)	Tm
XM_006347752.2	StEf1α	GGTTGTATCTCTTCCGATAAAGGC	GGTTGTATCTCTTCCGATAAAGGC	132	60
HS106768.1	StHSP90	CAGTGGTATCAACGCAGAGTAA	TCCTTCACAGACTTGTCATTCTT	107	62
Z11982.1	StHSP70	GGCATTGATCTTGGTACAACTTAT	GGAGTTGTTCTGTTGCCTTG	95	61
JX576239	StsHSP20	GGAGAGAGGAATGTGGAGAAAG	CGCATTCTCCGGAAGTCTAAA	102	62
XM_006341106.1	StHSFA3	CAGCTTTGTTCGACAGCTTAATAC	CAAATGCCTCTTCCCTCTCAA	100	62
XM_015308529.1	StP5CS	TGCAATGCAATGGAAACGCT	ACAATTTCCACGGTGCAAGC	194	60
AY442179	StCAT	CCATGCTGAGGTGTATCCTATTC	CCTTTCTCCTGGTTGCTTGA	100	62
AF354748	StSOD	CATTGGAAGAGCTGTTGTTGTT	ATCCTTCCGCCAGCATTT	96	62
XM_006362636.2	StPOD	AGATGTTGTGGCCATGTCTGG	GCTTGTGTTGAAGGATGGAGC	118	60
XM_015312004.1	StMAPK1	CTTATGGCATCGTTTGTGCCG	TGTCATGACCCATGTGACGAA	144	60
NM_001288519.1	StMAPK3	TGGTCTGTGGGTTGCATCTT	GGAGTGCCAAGAAGCTCAGT	107	60
NM_001288256.1	StMAPK4	TACGGAATCGTCTGCTGTGC	TCCTCTCTGTCTGGTGGTCTT	188	60
NM_001318567.1	StMAPK6	ACAACCCCTTTTTCCTGGCA	GTTGCCTTGGGTACCTTGGA	149	60
NM_001287969.1	StMAPK7	TACGGCCTTGTATGTGCTGC	GTTTTCGTGGTCCAAGTGCC	144	61
XM_006340601.2	StMAPK8	TGCGGCATATAAGGCATGAGA	AGCCACGAAGGAGCTGAAAA	189	60
XM_006341910.2	StMAPK9	AAGCTCCTTCGCCACCTAAG	CAGACCTCGAAGCAACTGGA	198	60
XM_006359965.2	StMAPK10	TGCATTTTCGCGGAGGTACT	GCCTTCTCATTACGAACCCCA	134	60
XM_006348278.2	StMAPK11	CGTCTCTCTCCCGCGATCTA	GCCAGCAGTTGCCTTTTCTG	106	60
XM_006348888.2	StMAPK12	CCACTGCTGAAGAGGCACTA	CCTTCGTCACTCTTCGCCTC	123	59
XM_006339355.2	StMAPK13	ATCATCCCTGCCTAGGGCTT	CTTGCAGCACCACCTTGAAC	189	60
XM_006360347.2	StMAPK14-1	CCTCATTGCCCAGAGAACGA	GGTGTACTATGGCCAGCCTC	190	60
XM_006344181.2	StMAPK14-2	GTCATTAAGGCCAACGGCAG	TGGAACACGTTTGCTGTGTG	101	59
XM_006365344.2	StMAPK16-1	TGGGTTTATGTACCCAAGTGGT	CATGCTGCCTCTGAAGTGGA	107	59
XM_006367486.2	StMAPK16-2	CGTGAACCATGTAGACCACCA	ACCGATCAACACCACTTGGG	187	60

### H_2_O_2_, proline, and MDA contents assay

H_2_O_2_ content, proline content, and MDA content was determined according to our previous methods (Zhu et al., 2021b).

### Chlorophyll content assay

Chlorophyll content in potato leaves was examined using the commercial chlorophyll assay kit according to the manuscript’s instruction (Item No. Cat#BC0990; Solarbio, Beijing, China). Fresh potato leaves were collected and washed in distilled water. After draining the surface of the leaves, the midrib of the leaves was removed and cut into pieces. Approximately 0.1 g of leaves were weighted and ground thoroughly in 1 mL water in the dark, which were then entirely transferred into a 10-mL volumetric flsk, diluted with water to volume, and mixed. The volumetric flsk was maintained in the dark for 3 h. The absorbance of the supernatant was measured at a wavelength of 663 nm and 645 nm using a spectrophotometer model 552 (Perkin Elmer, Shelton, CT, USA).

### Ion leakage assay

The top third functional leaves fully expanded were taken from the transgenic or non-transgenic potato plants at the same position. The leaves were washed with distilled water twice to remove any electrolytes adhering to the leaves or in the cut ends of the tissues. The water on the surface of the leaves were absorbed using filter paper. The leaf discs were punched with a 10 mm-punch from the potato leaves avoiding the central veins. The leaves were placed in 0.1 mol/L of mannitol aqueous solution and shaken at 100 times/min for 2 hours. The relative electrolyte leakage was examined using a conductometer (DDS-11A, Shanghai Scientific Instruments, Shanghai, China). The initial conductivity of the solution was recorded as L1. The solution was next boiled for 10 min and then cooled to 20°C before the determination of the final conductivity L2. The ion leakage was indicated as the ratio of L1 to L2.

### Activity assay of CAT, SOD, and POD

The activity of catalase (CAT), superoxide dismutase (SOD), and peroxidase (POD) was determined according to our previous methods (Zhu et al., 2021b).

### Net photosynthetic rate, transpiration rate and stomatal conductance assay

The third leaf from the plant top was collected when it fully expands during 9:30-11:30. Net photosynthetic rate, transpiration rate and stomatal conductance were examined using a portable photosynthetic LI-6400XT system (Li-COR, Lincoln, NE, USA). The photon flux density was set as 1,500 μmol·m^-2^·s^-1^. The relative humidity in leaf chamber was 50%-70%. CO_2_ concentration was 400 μmol/mol.

### Statistical analysis

All examinations were performed with three biological replicates and three technological replicates. Data are expressed as the mean ± standard deviation. Statistical analysis was performed with IBM SPSS 19.0 Statistical Software (IBM, Chicago, IL, USA) and GraphPad Prism Software (GraphPad, San Diego, CA, USA). Heatmap was prepared with R-software. Multiple comparison was analyzed by one-way ANOVA with Tukey test, Dunnett’s T3 or Sidak’s test for *post-hoc* analysis.

## Results

### Expression profile of StMAPKs in potato plants under heat stresses

**Figure 1:**
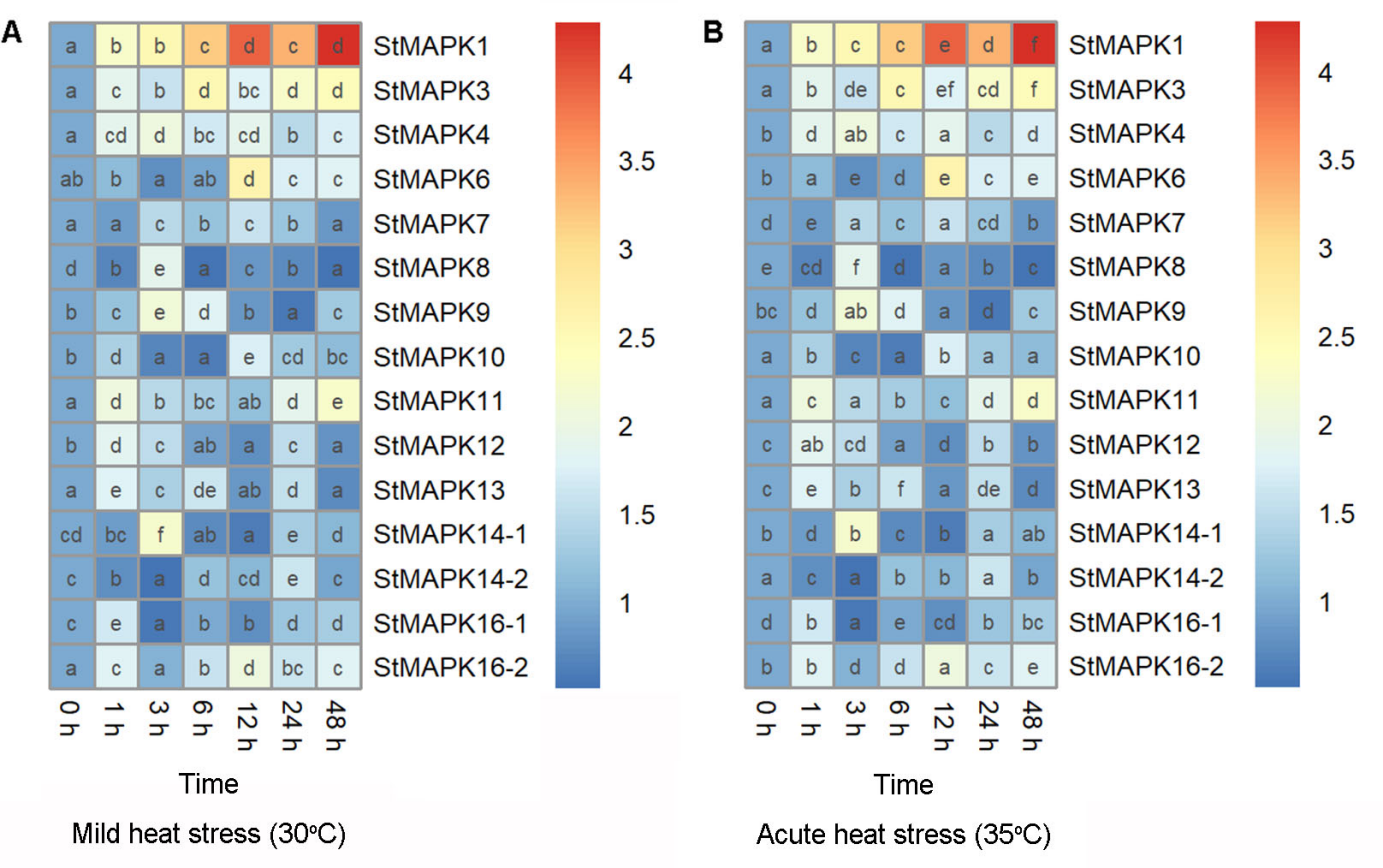
Heatmap showing mRNA expression of *StMAPK* gene family. Potato leaves were collected 0, 1, 3, 6, 12, 24, and 48 hours after cultivation under mild heat stress (30°C) **(A)** or wild heat stress (35°C) **(B)**. Data are mean ± standard deviation (n = 9). Different letters indicate significant difference (P < 0.05) among different samples by one-way ANOVA with Tukey test or Dunnett’s T3 for *post-hoc* analysis.

Potato plants were cultivated under mild (30°C) and acute (35°C) heat stresses, followed by examination of mRNA expression of *StMAPKs* (*StMAPK1*, *StMAPK3*, *StMAPK4*, *StMAPK6*, *StMAPK7*, *StMAPK8*, *StMAPK9*, *StMAPK10*, *StMAPK11*, *StMAPK12*, *StMAPK13*, *StMAPK14-1*, *StMAPK14-2*, *StMAPK16-1* and *StMAPK16-2*) (**Figure 1**). Transcript levels of *StMAPK* genes were found to be altered in response to mild (**Figure 1A**) and acute heat stresses (**Figure 1B**). Under heat stress conditions, *StMAPK1* and *StMAPK3* were induced to be expressed 1-48 h after heat stress (30°C and 35°C) treatment and peaked at 48 h and 6 h, respectively, with a 4.3-fold and 2.5-fold increase in expression, respectively (**Figure 1**). Notably, *StMAPK1* transcript levels were maintained at higher levels (expression increased more than 3-fold) at 6 h, 12 h, 24 h and 48 h of heat stress treatment (P < 0.05). The results indicated that heat stress at 30°C and 35°C induced a sustained and stable high expression of *StMAPK1*. Therefore, we focused on *StMAPK1* and studied its molecular function in the downstream experiment.

### StMAPK1 affected the physiological characteristics and phenotypes of potato plants under heat stresses

**Figure 2:**
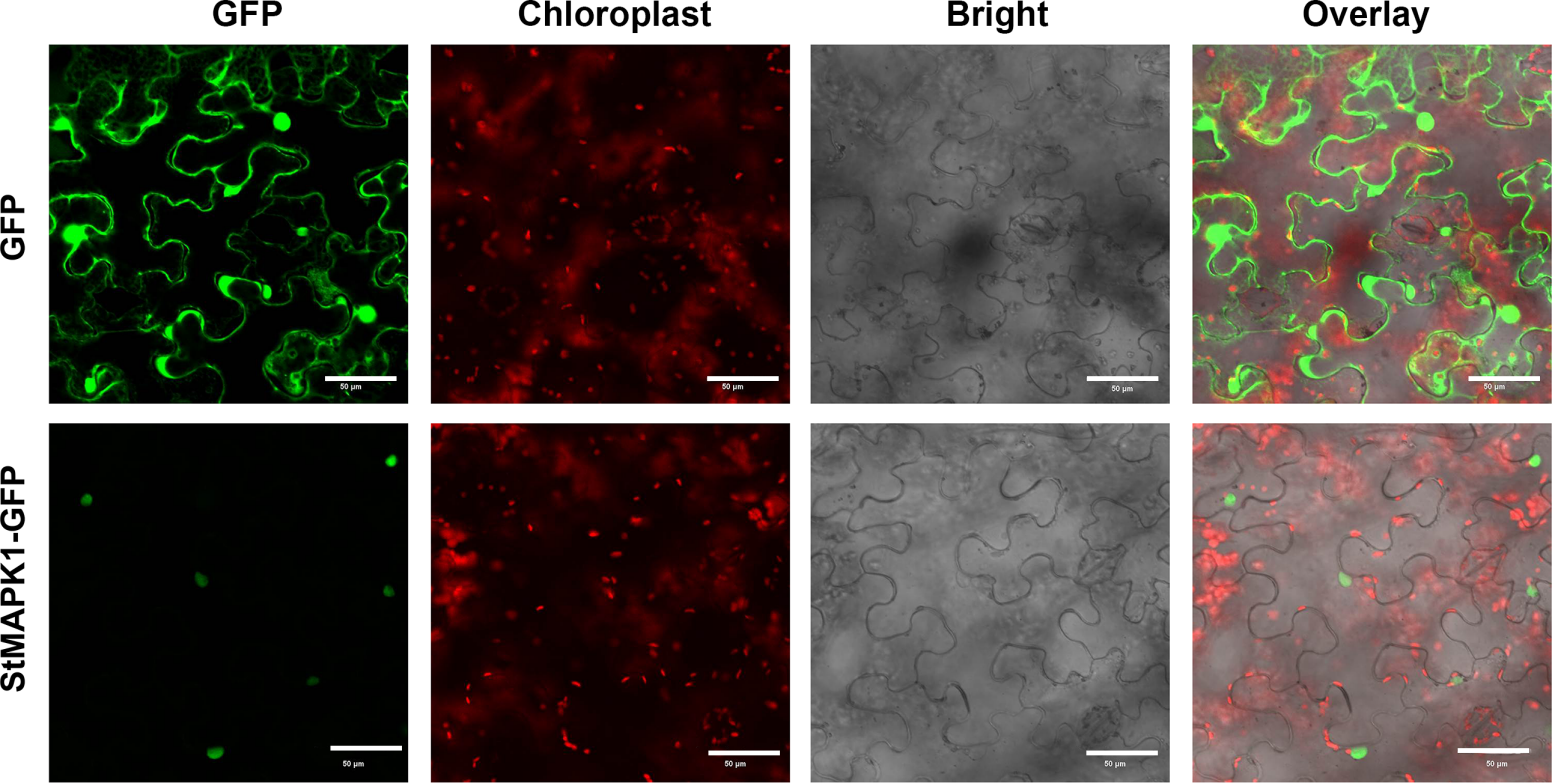
Subcellular localization of StMAPK1-GFP fusion protein. Confocal microscopy pictures showing the subcellular localization of StMAPK1-GFP in *Nicotiana tabacum* cells. StMAPK1-GFP construct was introduced into *Nicotiana tabacum* cells through agroinfiltration. The pictures were captured 48 h after infiltration. GFP served as a control. Scale bars = 50 μm.

**Figure 3:**
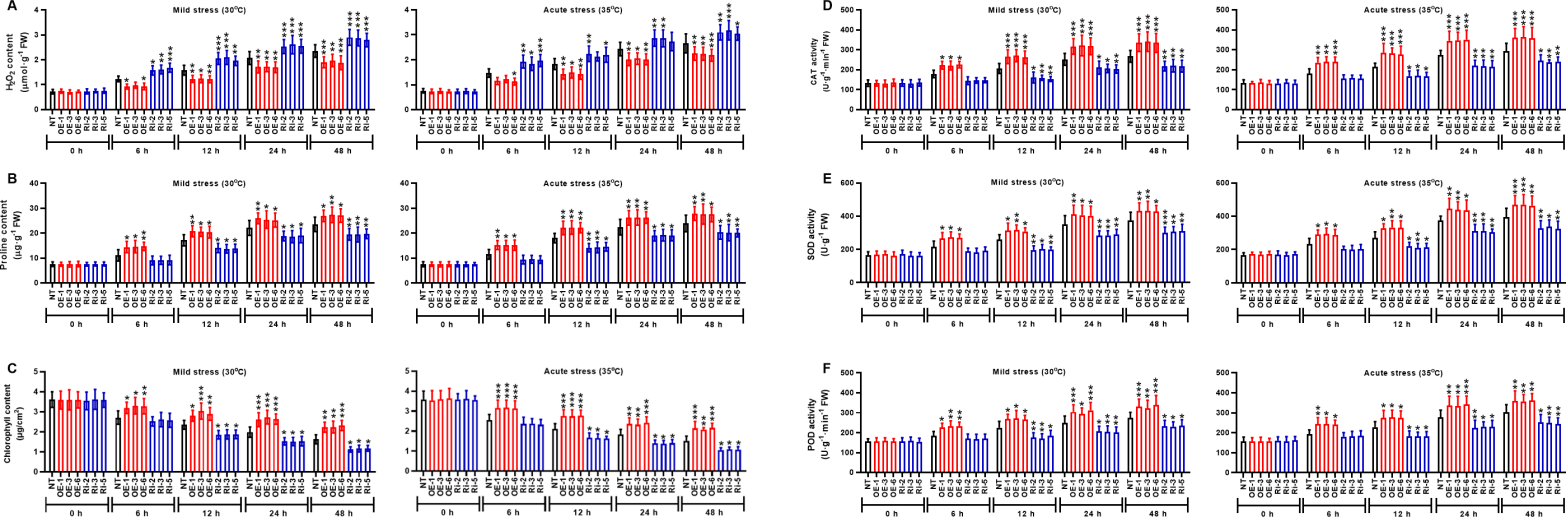
*StMAPK1* gene overexpression increased the contents of proline and chlorophyll and activities of CAT, SOD, and POD while decreased H_2_O_2_ content in response to heat stresses, **(A)** H_2_O_2_ content, **(B)** proline content, **(C)** chlorophyll content, **(D)** CAT activity, **(E)** SOD activity and **(F)** POD activity in potato leaves were examined 0, 6, 12, 24, and 48 hours after exposure to mild heat stress (30°C) and wild heat stress (35°C). Data are mean ± standard deviation (n = 9). *P < 0.05, **P < 0.01, ***P < 0.001 (OE or Ri groups compared to NT group, two-way ANOVA corrected by Sidak’s multiple comparisons test). Potato plants transfected with OE-1, OE-3 and OE-6 highly expressed *StMAPK1* mRNA; Potato plants in Ri-2, Ri-3 and Ri-5 lowly expressed *StMAPK1* mRNA.

MAPK cascade transduces the heat signals from the extracellular space to the nucleus, and MAPK is involved in plant signal transduction. The subcellular localization of MAPK1 protein was predicted using the online tool WoLFPSORT (https://wolfpsort.hgc.jp/). The results showed that MAPK1 protein locates in the nucleus. To ascertain the subcellular localization of *StMAPK1*, GFP-tagged StMAPK1 protein was imaged by confocal fluorescent microscopy. It was observed that GFP-StMAPK1 can localize to the nucleus (**Figure 2**). *StMAPK1* expression in the transgenic plants (OE and RNAi groups) was shown in **Additional Figure 1**. *StMAPK1* overexpression decreased the generation of H_2_O_2_ under mild and acute heat stresses compared to the NT group (P < 0.05, P < 0.01, P < 0.001) (**Figure 3A**). In contrast, *StMAPK1* knockdown induced the accumulation of H_2_O_2_ compared to the NT group (P < 0.05, P < 0.01, P < 0.001). *StMAPK1* overexpression elevated the contents of proline and chlorophyll compared to the NT group (P < 0.05, P < 0.01, P < 0.001) (**Figure 3B** and **Figure 3C**). However, *StMAPK1* knockdown leads to the decreased content of proline and chlorophyll when compared to the NT group (P < 0.05, P < 0.01, P < 0.001). The activity of CAT, SOD and POD was significantly enhanced in *StMAPK1*-transgenic plants compared to the NT group (P < 0.05, P < 0.01, P < 0.001) (**Figures 3D-F**). By comparison, potato plants with *StMAPK1* knockdown were observed with decreased activities of CAT, SOD, and POD (P < 0.05, P < 0.01, P < 0.001). Additionally, we observed that *StMAPK1* knockdown retarded the growth of potato plants in terms of plant height, fresh weight, dry weight, root fresh weight and root dry weight (**Additional Figure 2** and **Additional Table 1**). However, the potato plants were cultured at 35°C for 48 h and then transferred to 20°C for 72 h. The growth state of *StMAPK1* overexpressed plants was maintained, while the growth of *StMAPK1* low-expressed plants was delayed. Additionally, compared to the NT plants, both OE and Ri plants were observed with yellow leaves.

### StMAPK1 modulates photosynthesis and membrane integrity under heat stresses

**Figure 4:**
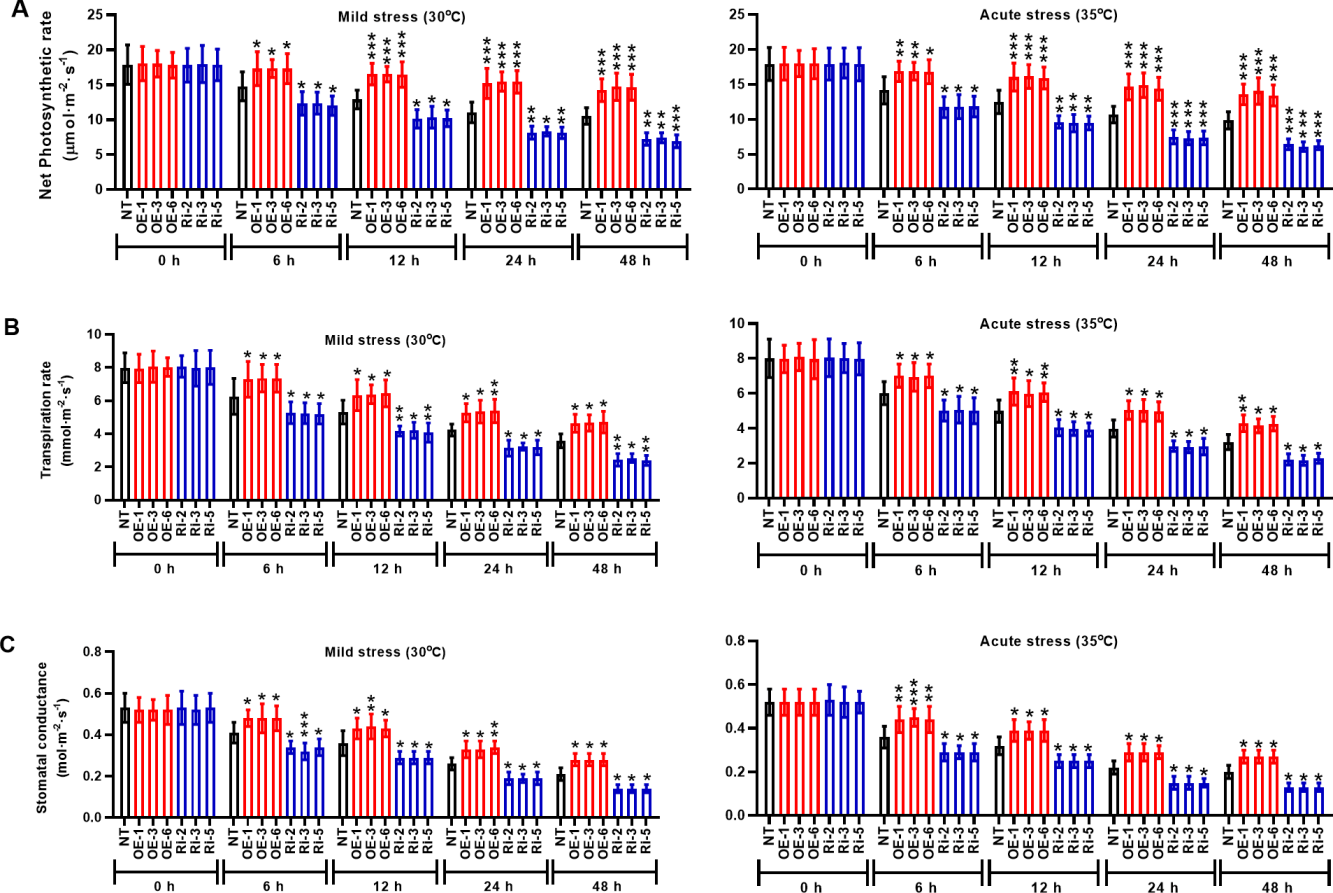
*StMAPK1* gene overexpression elevated the photosynthesis of potato leaves in response to heat stresses. **(A)** Net photosynthetic rate, **(B)** transpiration rate, and **(C)** stomatal conductance of potato leaves were examined 0, 6, 12, 24, and 48 hours after exposure to mild heat stress (30°C) and wild heat stress (35°C). Data are mean ± standard deviation (n = 9). *P < 0.05, **P < 0.01, ***P < 0.001 (OE or Ri groups compared to NT group, two-way ANOVA corrected by Sidak’s multiple comparisons test). Potato plants transfected with OE-1, OE-3 and OE-6 highly expressed *StMAPK1* mRNA; Potato plants in Ri-2, Ri-3 and Ri-5 lowly expressed *StMAPK1* mRNA.

**Figure 5:**
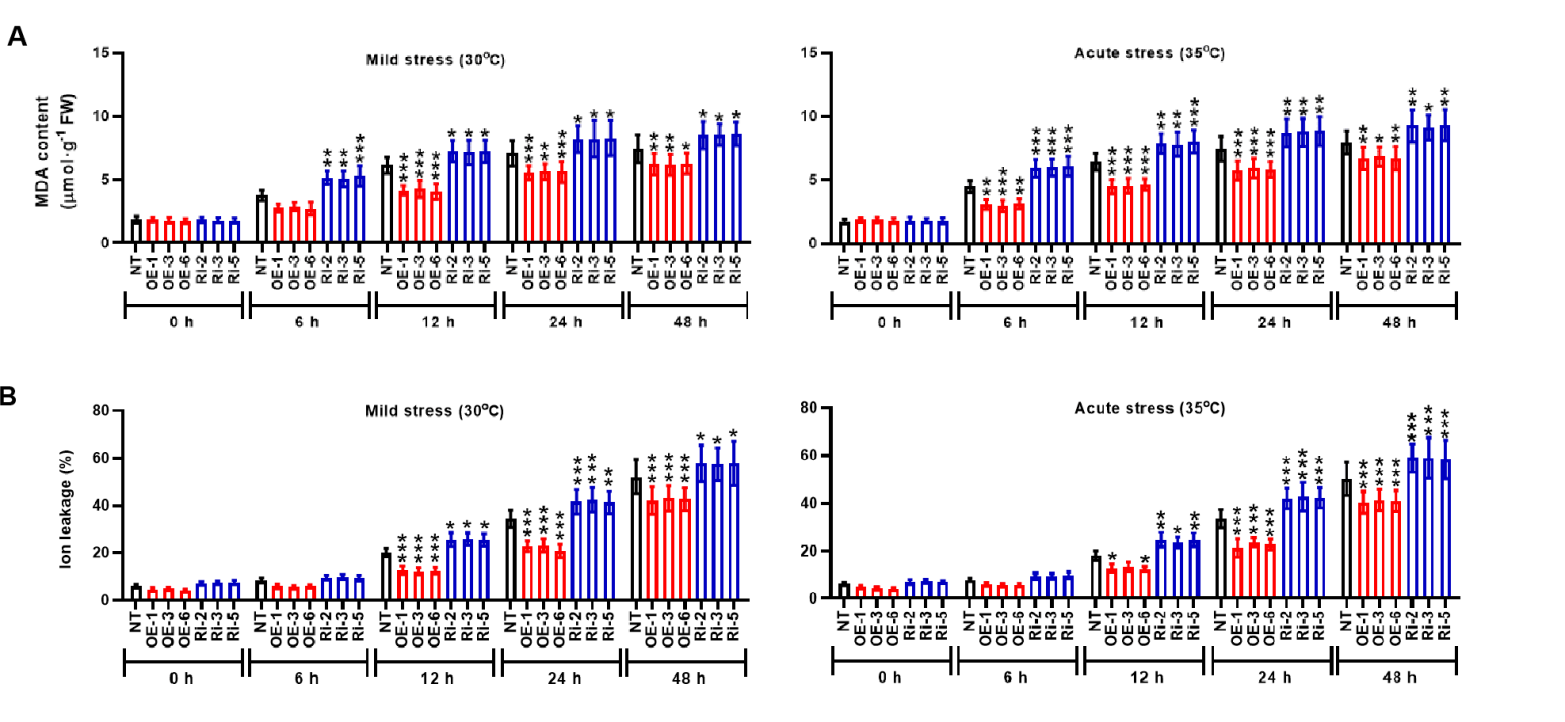
*StMAPK1* gene overexpression retains the cell membrane integrity under heat stress. **(A)** MDA content and **(B)** ion leakage of potato leaves were examined 0, 6, 12, 24, and 48 hours after exposure to mild heat stress (30°C) and wild heat stress (35°C). Data are mean ± standard deviation (n = 9). *P < 0.05, **P < 0.01, ***P < 0.001 (OE or Ri groups compared to NT group, two-way ANOVA corrected by Sidak’s multiple comparisons test). Potato plants transfected with OE-1, OE-3 and OE-6 highly expressed *StMAPK1* mRNA; Potato plants in Ri-2, Ri-3 and Ri-5 lowly expressed *StMAPK1* mRNA.

To identity the molecular mechanisms mediated by *StMAPK1* gene, we examined the photosynthesis and membrane integrity of potato plants under mild and acute heat stresses. It was noted that *StMAPK1* retained the net photosynthetic rate (**Figure 4A**), transpiration rate (**Figure 4B**) and stomatal conductance (**Figure 4C**) under mild and acute heat stresses (P < 0.05, P < 0.01, P < 0.001). *StMAPK1* knockdown conversely decreased the net photosynthesis, transpiration rate and stomatal conductance compared to the NT group (P < 0.05, P < 0.01, P < 0.001). We next quantitatively evaluated the thermo-tolerance of potato plants. The membrane integrity was assessed by measuring ion leakage and MDA content. Heat stresses increased MDA content and facilitated ion leakage, while *StMAPK1* overexpression significantly prevented the outcome (P < 0.05, P < 0.01, P < 0.001) (**Figures 5A, B**). On the contrary, *StMAPK1* knockdown had the opposite effects on the membrane integrity compared to the NT group (P < 0.05, P < 0.01, P < 0.001).

### StMAPK1 regulates the expression of stress response genes (StP5CS, StCAT, StSOD, and StPOD) in potato plants

**Figure 6:**
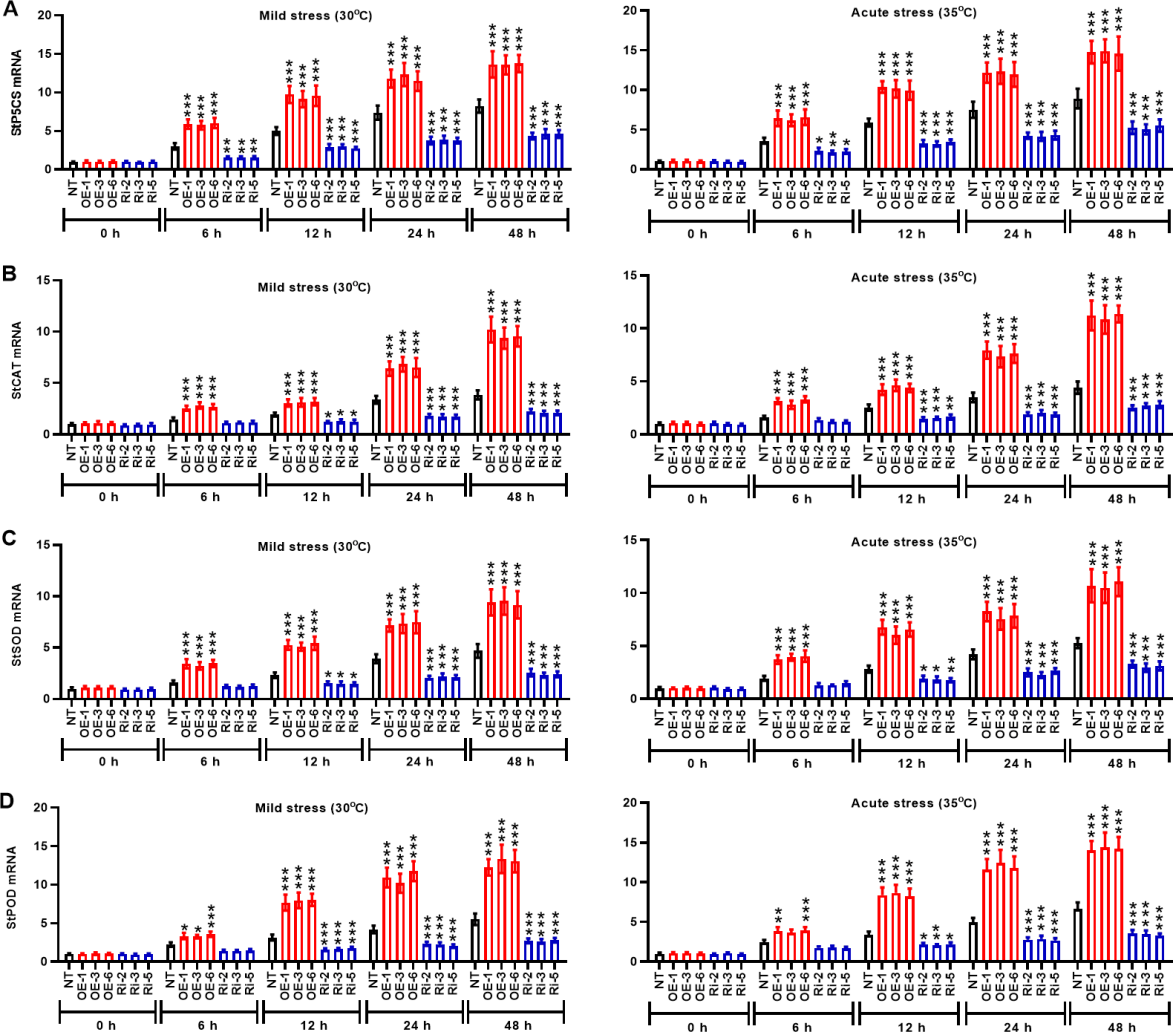
*StMAPK1* gene overexpression elevated mRNA expression of stress response genes (*StP5CS*, *StCAT*, *StSOD*, and *StPOD*) in potato plants in response to heat stress. Transcription levels of **(A)** *StP5CS*, **(B)** *StCAT*, **(C)** *StSOD*, and **(D)** *StPOD* in potato leaves were examined 0, 6, 12, 24, and 48 hours after exposure to mild heat stress (30°C) and wild heat stress (35°C). Data are mean ± standard deviation (n = 9). *P < 0.05, **P < 0.01, ***P < 0.001 (OE or Ri groups compared to NT group, two-way ANOVA corrected by Sidak’s multiple comparisons test). Potato plants transfected with OE-1, OE-3 and OE-6 highly expressed *StMAPK1* mRNA; Potato plants in Ri-2, Ri-3 and Ri-5 lowly expressed *StMAPK1* mRNA.

Under mild and acute heat stresses, *StMAPK1* gene was involved in the regulation of the transcription of stress response genes such as *StP5CS*, *StCAT*, *StSOD*, and *StPOD*. Mild and acute heat stress induced the transcription of stress response genes *StP5CS* (**Figure 6A**), *StCAT* (**Figure 6B**), *StSOD* (**Figure 6C**), and *StPOD* (**Figure 6D**) with the extension of incubation time (P < 0.05, P < 0.01, P < 0.001). In *StMAPK1*-transgenic potato plants, mRNA expression of the examined stress response genes was significantly increased compared to the NT group (P < 0.05). By contrast, the transcript levels of *StP5CS*, *StCAT*, *StSOD*, and *StPOD* genes were significantly lower in the *StMAPK1* gene knockdown plants than those of the NT plants (P < 0.05). Hence, we hypothesized that *StMAPK1* gene may affect the heat response of potato plants by increasing the transcription of *StP5CS*, *StCAT*, *StSOD*, and *StPOD* genes.

### StMAPK1 induces the expression of heat stress genes (StHSP90, StHSP70, StHSP20, and StHSFA3) in potato leaves in response to heat stress

**Figure 7:**
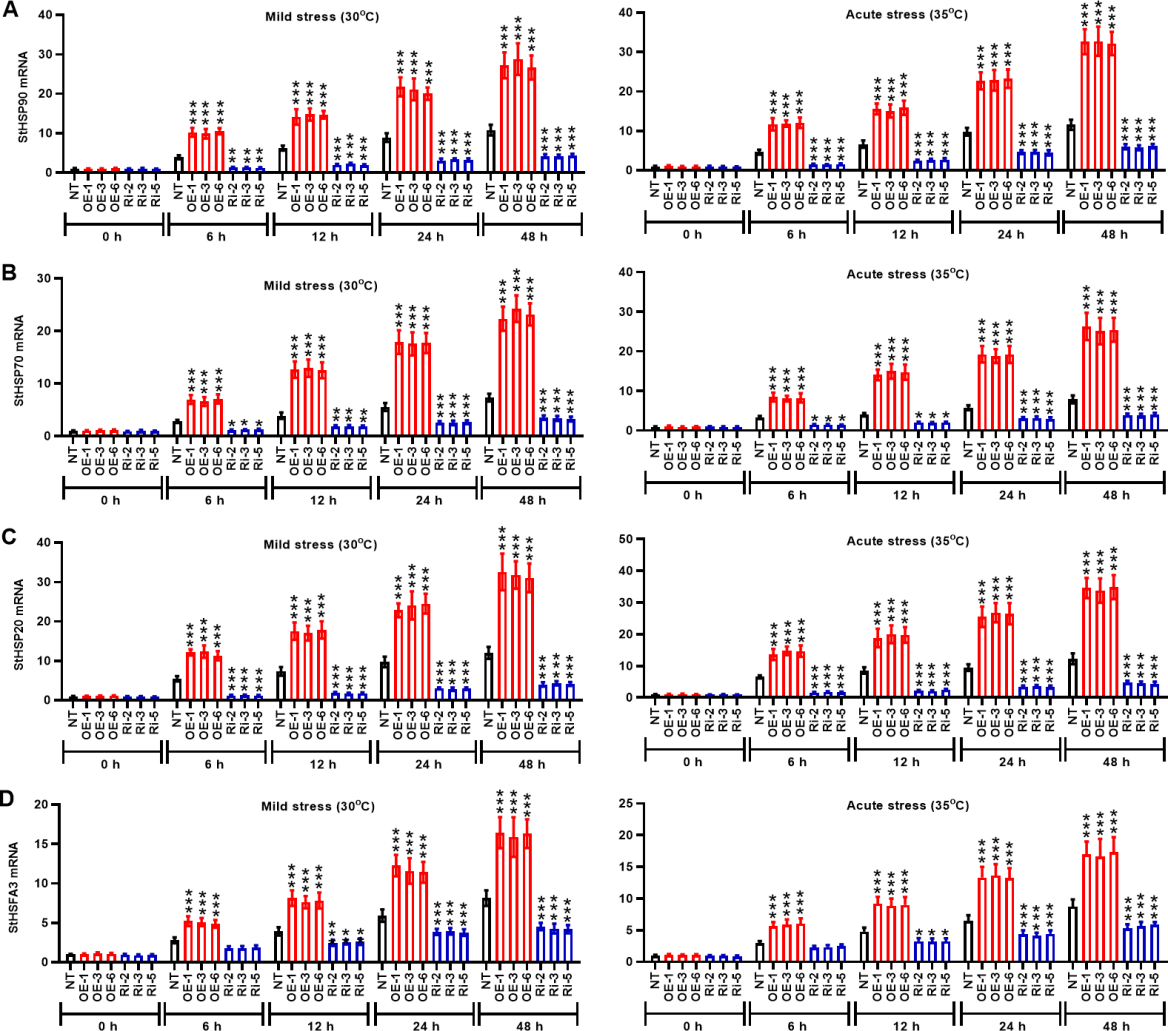
*StMAPK1* gene overexpression enhances mRNA expression of heat response genes (*StHSP90*, *StHSP70, StHSP20*, and *StHSFA3*) in potato plants. Transcription levels of **(A)** *StHSP90*, **(B)** *StHSP70*, **(C)** *StHSP20*, and **(D)** *StHSFA3* in potato leaves were examined 0, 6, 12, 24, and 48 hours after exposure to mild heat stress (30°C) and wild heat stress (35°C). Data are mean ± standard deviation (n = 9). *P < 0.05, **P < 0.01, ***P < 0.001 (OE or Ri groups compared to NT group, two-way ANOVA corrected by Sidak’s multiple comparisons test). Potato plants transfected with OE-1, OE-3 and OE-6 highly expressed *StMAPK1* mRNA; Potato plants in Ri-2, Ri-3 and Ri-5 lowly expressed *StMAPK1* mRNA.

The kinetics of heat stress response were evaluated in potato plants of the OE group and Ri group in terms of the mRNA expression of *StHSP90*, *StHSP70*, *StHSP20*, and *StHSFA3* genes). During the entire period (0 h to 48 h) of heat stress, the mRNA levels of *StHSP90* (**Figure 7A**), *StHSP70* (**Figure 7B**), *StHSP20* (**Figure 7C**), and *StHSFA3* (**Figure 7D**) genes were prominently increased in the NT plants compared to the initial time (0 h) (P < 0.05). The mRNA expression of heat stress genes in the OE plants was significantly higher than that in the NT group at the same time after heat stress induction (P < 0.05). However, potato plants in the Ri group conversely exhibited low expression of heat stress genes *StHSP90*, *StHSP70*, *StHSP20*, and *StHSFA3* (P < 0.05) compared to the NT plants. Thus, *StMPAK1* may mediate the heat stress response of potato plants by increasing the transcription of heat stress genes *StHSP90*, *StHSP70*, *StHSP20*, and *StHSFA3*.

## Discussion

Potato plants exposed to high temperatures (above 30°C) have been reported to produce and maintain relatively large canopies, but fail to generate tubers (Ewing, 1981; Singh et al., 2020). Zhanjiang (110°22′6″E, 21°15′55″N) is located to the south of the Tropic of Cancer, in the area of influence of the southern subtropical monsoon. In Zhanjiang, potato is grown in December (15-21°C) and harvested in March (19-24°C). However, as global warming continues to increase, Zhanjiang has recorded temperatures in excess of 30°C in March. Therefore, it is of significance to determine plant morphology, growth status, physiological indexes, and molecular responses of potato plants when exposed to temperature above 30°C.

In this study, we examined the mRNA level of potential candidate *MAPKs* family in potato plants exposed to mild heat stress (30°C) and acute heat stress (35°C). Among these genes, *MAPK1* gene exhibited a steady increase in transcript level in potato leaves under heat stress conditions, indicating that *MAPK1* gene may be in relevance with the sensitivity to high temperature and modulate the acquired thermo-tolerance of potato plants. The serine/threonine protein kinase family of mitogen-activated protein kinases are highly conserved signaling transduction modules in signaling transduction processes through MAPK cascades (Bharti et al., 2021; Zhang and Zhang, 2022). As the temperature elevates from the optimal threshold to the limit temperature, MAPK protein activates downstream kinases, enzymes, transcription factors and other response factors, which accomplishes the transmission of extracellular environmental signals into cells (Bharti et al., 2021; Lin et al., 2021; Mo et al., 2021). Here, we found that StMAPK1 protein located to the cell nucleus, which may phosphorylate other specific transcription factors for functioning in further signal transmission.

At the biochemical level, plants alter the generation of compatible solutes such as H_2_O_2_ and proline to maintain redox balance and turgor pressure (Sarker and Oba, 2018; Forlani et al., 2019). Our results demonstrated that genetic manipulation of the expression of *MAPK1* gene predominantly enhances the tolerance to heat stresses in potato plant by altering several physiological indexes including H_2_O_2_ content, proline content, and chlorophyll content. H_2_O_2_is a well-recognized product produced in photosynthetic tissues under oxidative stress (Hirt, 2000). *AtMAPKKKs* regulate H_2_O_2_ signal transduction through activating *MAPKs* such as *AtMAPK3* and *AtMAPK6* (Hirt, 2000). Maize *MAPK1* plays a positive role in Arabidopsis, response to heat stress (Wu et al., 2015). Chlorophyll plays a major role in the process of photosynthesis, which is affected by high temperature (Singh et al., 2020). Our results suggested that heat stress decreased total chlorophyll content, however, upregulation of *StMAPK1* ameliorated the loss of chlorophyll. *StMAPK1* gene played an important role in plant growth in response to mild and acute heat stress, which was evidenced by the increased activities of CAT, SOD, and POD. In response to heat stress, MAPK pathway triggers the activation of its downstream redox-sensitive transcription factors that are involved in the generation of multitudinous antioxidant enzymes such as CAT, SOD and POD (Delaunay et al., 2002) (Gill and Tuteja, 2010) (Delaunay et al., 2002; Dąbrowska et al., 2007; Dąbrowska et al., 2007; Gill and Tuteja, 2010).

The prolonged increase in temperature or extreme high temperature moderates the photosynthetic efficiency, which may be ascribed to the denaturation of the active enzymes or lipid peroxidation of chlorophyll (Killi et al., 2020; Xu et al., 2021). Here, we observed that *StMAPK1* gene is a positive regulator of photosynthesis in potato plants. Transgenic plants overexpressing *StMAPK1* exhibited increased net photosynthetic rate, transpiration rate and stomatal conductance. To maintain cellular homeostasis, plants have evolved mechanisms to maintain membrane stability (Conde et al., 2011). In this study, the available results indicated that *StMAPK1* decreased MDA content and reduced ion leakage, suggesting that *StMAPK1* maintained cell membrane integrity and inhibit cell injuries in response to extreme high temperature. The severely affected photosynthesis and heat stress-related injuries ultimately lead to growth inhibition and a decrease in crop yield.

In our study, *StMAPK1* gene was considered to affect mRNA expression of stress response genes (*StP5CS*, *StCAT*, *StSOD*, and *StPOD*) in potato plants. P5CS gene is confirmed to increase resistance of transgenic plants in response to heat, drought, salinity, which is accomplished via enhancing proline content (Amini et al., 2015). In particular, *StMAPK1* gene was involved in the regulation of heat response gene expression, such as *StHSP90*, *StHSP70*, *StHSP20*, and *StHSFA3* genes in potato plants. HSP90 and HSP70 belong to chaperone family involved in cellular homeostasis, which participate in the control of heat stress response by modulating heat stress transcription factors in plants (Hahn et al., 2011). Genome-wide analysis revealed that *StHSP20* gene is expressed in various tissues and organs, and its expression responds to heat, drought and salt stresses (Zhao et al., 2018). *HSFA3* encoding heat stress transcription factor HSFA3 is essential for plants to resist rapidly changing heat stresses and to enhance thermos-tolerance (Schramm et al., 2008). These results suggested that *StMAPK1* may be the upstream regulator of heat stress genes, which enlaces potato plants to adapt to or resist to high temperature stress.

## Conclusions

In this study, we described that *StMAPK* gene family is involved in responses to heat stresses. Several lines of evidence suggested that *MAPK1* gene is included in signaling heat stress in potato plants, resulting in the induction of physiological cellular responses. Genetic studies of *MAPK1* gene knockdown revealed its importance in the regulation of the expression of defense genes and heat stress response genes. Summarily, the study elucidates a molecular mechanism enabling potato plants rapidly respond to heat stress conditions.

## Data availability statement

The original contributions presented in the study are included in the article/supplementary material. Further inquiries can be directed to the corresponding authors.

## Author contributions

XZ, HD, GZ, and YZ planned and designed the research. XZ, HD, GZ, CX, SC, and CZ collected the data. XZ, HD, HJ, ZC, and JT analyzed the data. XZ, HD, CX, and YZ drafted the manuscript. All authors have read and agreed to the published version of the manuscript. All authors contributed to the article.

## Supplementary material

Supplementary Figure 1 mRNA expression of *StMAPK1* in potato plants transfected with pBI121-EGFP plasmid carrying StMAPK1 protein-coding gene **(A)** and pART plasmid carrying anti-sense cDNA sequence of *StMAPK1* gene **(B)**. Data are mean ± standard deviation (n = 9). ***P < 0.001 (OE or RNAi compared to NC, two-way ANOVA corrected by Sidak’s multiple comparisons test).

Supplementary Figure 2 Effects of *StMAPK1* on potato phenotypes in response to heat stress. Phenotypic changes of potato plants cultivated 0 h, 12 h, 24 h, and 48 h after exposure to wild heat stress (35°C). The bottom group of potato pants was incubated at 35°C for 48 h, and then transferred to 20°C and cultivated for 72 h. Potato plants transfected with OE-1, OE-3 and OE-6 highly expressed *StMAPK1* mRNA; Potato plants in Ri-2, Ri-3 and Ri-5 lowly expressed *StMAPK1* mRNA.

## References

[B12] Almeselmani M. Deshmukh P. S. Sairam R. K Kushwaha S. R. Singh T. P. (2006). Protective role of antioxidant enzymes under high temperature stress. Plant Sci. 171 (3), 382–388. doi: 10.1016/j.plantsci.2006.04.009 22980208

[B44] Amini S. Ghobadi C. Yamchi A. (2015). Proline accumulation and osmotic stress: an overview of P5CS gene in plants. J. Plant Mol. Breed. 3 (2), 44–55.

[B8] Arreguin-Lozano B. Bonner J. (1949). Experiments on sucrose formation by potato tubers as influenced by temperature. Plant Physiol. 24 (4), 720. doi: 10.1104/pp.24.4.720 16654259 PMC437419

[B28] Bharti J. Sahil Mehta S. Ahmad S. Singh B. Padhy A. K.. (2021). Mitogen-activated protein kinase, plants, and heat stress. Harsh Environ. Plant Resilience: Mol. Funct. Aspects p, 323–354. doi: 10.1007/978-3-030-65912-7_13

[B2] Chen C. Jiang Y. van Groenigen K. J. Hungate B. A. Zhang J. Liu J.. (2020). Global warming and shifts in cropping systems together reduce china’s rice production. Global Food Secur. 24, 100359. doi: 10.1016/j.gfs.2020.100359

[B43] Conde A. Chaves M. M. Gerós H. (2011). Membrane transport, sensing and signaling in plant adaptation to environmental stress. Plant Cell Physiol. 52 (9), 1583–1602. doi: 10.1093/pcp/pcr107 21828102

[B37] Dąbrowska G. Kata A. Goc A. Szechyńska-Hebda M. Skrzypek E. (2007). Characteristics of the plant ascorbate peroxidase family. Acta Biologica Cracoviensia Ser. Botanica 49 (1), 7–17.

[B35] Delaunay A. Pflieger D. Barrault M.-B. Vinh J. Toledano M. (2002). A thiol peroxidase is an H2O2 receptor and redox-transducer in gene activation. Cell 111 (4), 471–481. doi: 10.1016/S0092-8674(02)01048-6 12437921

[B26] Ewing E. (1981). Heat stress and the tuberization stimulus. Am. Potato J. 58, 31–49. doi: 10.1007/BF02855378

[B13] Fogelman E. Oren‑Shamir M. Hirschberg J. Mandolino G. Parisi B. Ovadia R.. (2019). Nutritional value of potato (Solanum tuberosum) in hot climates: anthocyanins, carotenoids, and steroidal glycoalkaloids. Planta 249, 1143–1155. doi: 10.1007/s00425-018-03078-y 30603793

[B32] Forlani G. Trovato M. Funck D. Signorelli S. (2019). Regulation of proline accumulation and its molecular and physiological functions in stress defence. Osmoprotectant-mediated abiotic Stress tolerance plants: Recent Adv. Future Perspect. p, 73–97. doi: 10.1007/978-3-030-27423-8_3

[B36] Gill S. S. Tuteja N. (2010). Reactive oxygen species and antioxidant machinery in abiotic stress tolerance in crop plants. Plant Physiol. Biochem. 48 (12), 909–930. doi: 10.1016/j.plaphy.2010.08.016 20870416

[B45] Hahn A. Bublak D. Schleiff E. Scharf K.-D. (2011). Crosstalk between Hsp90 and Hsp70 chaperones and heat stress transcription factors in tomato. Plant Cell 23 (2), 741–755. doi: 10.1105/tpc.110.076018 21307284 PMC3077788

[B4] Handayani T. Gilani S. A. Watanabe K. N. (2019). Climatic changes and potatoes: how can we cope with the abiotic stresses? Breed. Sci. 69 (4), 545–563. doi: 10.1270/jsbbs.19070 31988619 PMC6977456

[B33] Hirt H. (2000). Connecting oxidative stress, auxin, and cell cycle regulation through a plant mitogen-activated protein kinase pathway. Proc. Natl. Acad. Sci. 97 (6), 2405–2407. doi: 10.1073/pnas.97.6.2405 10716978 PMC33972

[B41] Killi D. Raschi A. Bussotti F. (2020). Lipid peroxidation and chlorophyll fluorescence of photosystem II performance during drought and heat stress is associated with the antioxidant capacities of C3 sunflower and C4 maize varieties. Int. J. Mol. Sci. 21 (14), 4846. doi: 10.3390/ijms21144846 32659889 PMC7402356

[B11] Kim Y.-U. Seo B.-S. Choi D.-H. Ban H.-Y. Lee B.-W. (2017). Impact of high temperatures on the marketable tuber yield and related traits of potato. Eur. J. Agron. 89, 46–52. doi: 10.1016/j.eja.2017.06.005

[B3] Lee Y.-H. Sang W.-G. Baek J.-K. Kim J.-H. Shin P Seo M.-C.. (2020). The effect of concurrent elevation in CO2 and temperature on the growth, photosynthesis, and yield of potato crops. PloS One 15 (10), e0241081. doi: 10.1371/journal.pone.0241081 33085713 PMC7577495

[B22] Li G. Cao C. Yang H. Wang J. Wei W. Zhu D.. (2020). Molecular cloning and potential role of DiSOC1s in flowering regulation in davidia involucrata baill. Plant Physiol. Biochem. 157, 453–459. doi: 10.1016/j.plaphy.2020.11.003 33218844

[B20] Lin L. Wu J. Jiang M. Wang Y. (2021). Plant mitogen-activated protein kinase cascades in environmental stresses. Int. J. Mol. Sci. 22 (4), 1543. doi: 10.3390/ijms22041543 33546499 PMC7913722

[B14] Link V. Sinha A. K. Vashista P. Hofmann M. G. Proels R. K. Ehness R.. (2002). A heat-activated MAP kinase in tomato: a possible regulator of the heat stress response. FEBS Lett. 531 (2), 179–183. doi: 10.1016/S0014-5793(02)03498-1 12417308

[B24] Lu H. Klocko A. L. Brunner A. M. Ma C. Magnuson A. C. Howe G. T.. (2019). RNA Interference suppression of AGAMOUS and SEEDSTICK alters floral organ identity and impairs floral organ determinacy, ovule differentiation, and seed-hair development in populus. New Phytol. 222 (2), 923–937. doi: 10.1111/nph.15648 30565259 PMC6590139

[B21] Majeed Y. Zhu X. Zhang N. Rasheed A. Tahir M. M. Si H. (2022). Functional analysis of mitogen-activated protein kinases (MAPKs) in potato under biotic and abiotic stress. Mol. Breed. 42 (6), 31. doi: 10.1007/s11032-022-01302-y 37312964 PMC10248695

[B17] Majeed Y. Zhu X. Zhang N. ul-Ain N. Raza A. Haider F. U.. (2023). Harnessing the role of mitogen-activated protein kinases against abiotic stresses in plants. Front. Plant Sci. 14, 932923. doi: 10.3389/fpls.2023.932923 36909407 PMC10000299

[B9] Menzel C. (1983). Tuberization in potato at high temperatures: interaction between shoot and root temperatures. Ann. Bot. 52 (1), 65–69. doi: 10.1093/oxfordjournals.aob.a086553

[B30] Mo S. Qian Y. Zhang W. Qian L. Wang Y. Cailin G.. (2021). Mitogen-activated protein kinase action in plant response to high-temperature stress: a mini review. Protoplasma 258, 477–482. doi: 10.1007/s00709-020-01603-z 33392739

[B10] Rykaczewska K. (2015). The effect of high temperature occurring in subsequent stages of plant development on potato yield and tuber physiological defects. Am. J. Potato Res. 92, 339–349. doi: 10.1007/s12230-015-9436-x

[B31] Sarker U. Oba S. (2018). Drought stress effects on growth, ROS markers, compatible solutes, phenolics, flavonoids, and antioxidant activity in amaranthus tricolor. Appl. Biochem. Biotechnol. 186, 999–1016. doi: 10.1007/s12010-018-2784-5 29804177

[B47] Schramm F. Larkindale J. Kiehlmann E. Ganguli A. Englich G. Vierling E.. (2008). A cascade of transcription factor DREB2A and heat stress transcription factor HsfA3 regulates the heat stress response of arabidopsis. Plant J. 53 (2), 264–274. doi: 10.1111/j.1365-313X.2007.03334.x 17999647

[B23] Si H.-J. Xie C.-H. Liu J. (2003). An efficient protocol for agrobacterium-mediated transformation with microtuber and the introduction of an antisense class I patatin gene into potato. Acta Agronomica Sin. 29 (6), 801–805.

[B27] Singh B. Kukreja S. Goutam U. (2020). Impact of heat stress on potato (Solanum tuberosum l.): present scenario and future opportunities. J. Hortic. Sci. Biotechnol. 95 (4), 407–424.

[B15] Suri S. S. Dhindsa R. S. (2008). A heat-activated MAP kinase (HAMK) as a mediator of heat shock response in tobacco cells. Plant Cell Environ. 31 (2), 218–226. doi: 10.1111/j.1365-3040.2007.01754.x 17996015

[B6] Wolf S. Olesinski A. A. Rudich J. Marani A. (1990). Effect of high temperature on photosynthesis in potatoes. Ann. Bot. 65 (2), 179–185. doi: 10.1093/oxfordjournals.aob.a087922

[B5] Wolf S. Marani A. Rudich J. (1991). Effect of temperature on carbohydrate metabolism in potato plants. J. Exp. Bot. 42 (5), 619–625. doi: 10.1093/jxb/42.5.619

[B19] Wu L. Zu X. Zhang H. Wu L. Xi Z. Chen Y. (2015). Overexpression of ZmMAPK1 enhances drought and heat stress in transgenic arabidopsis thaliana. Plant Mol. Biol. 88 (4), 429–443. doi: 10.1007/s11103-015-0333-y 26008677

[B42] Xu C. Wang M. T. Yang Z. Q. Han W Zheng S. H. (2021). Effects of high temperature on photosynthetic physiological characteristics of strawberry seedlings in greenhouse and construction of stress level. Ying Yong Sheng tai xue bao= J. Appl. Ecol. 32 (1), 231–240. 10.13287/j.1001-9332.202101.028 33477231

[B7] Yubi Y. Jun L. Haiyang N. Xiuyun Z. (2019). Collaborative influence of elevated CO2 concentration and high temperature on potato biomass accumulation and characteristics. Open Chem. 17 (1), 728–737. doi: 10.1515/chem-2019-0085

[B1] Zandalinas S. I. Fritschi F. B. Mittler R. (2021). Global warming, climate change, and environmental pollution: recipe for a multifactorial stress combination disaster. Trends Plant Sci. 26 (6), 588–599. doi: 10.1016/j.tplants.2021.02.011 33745784

[B16] Zaynab M. Hussain A. Sharif Y. Fatima M. Sajid M. Rehman N.. (2021). Mitogen-activated protein kinase expression profiling revealed its role in regulating stress responses in potato (Solanum tuberosum). Plants (Basel) 10 (7), 1371. doi: 10.3390/plants10071371 34371574 PMC8309457

[B29] Zhang M. Zhang S. (2022). Mitogen-activated protein kinase cascades in plant signaling. J. Integr. Plant Biol. 64 (2), 301–341. doi: 10.1111/jipb.13215 34984829

[B46] Zhao P. Wang D. Wang R. Kong N. Zhang C. Yang C.. (2018). Genome-wide analysis of the potato Hsp20 gene family: identification, genomic organization and expression profiles in response to heat stress. BMC Genomics 19 (1), 61. doi: 10.1186/s12864-018-4443-1 29347912 PMC5774091

[B18] Zhu X. Zhang N. Liu X Li S. Yang J. Hong X.. (2021a). Mitogen-activated protein kinase 11 (MAPK11) maintains growth and photosynthesis of potato plant under drought condition. Plant Cell Rep. 40, 491–506. doi: 10.1007/s00299-020-02645-6 33388892

[B25] Zhu X. Hong X. Liu X. Li S. Yang J. Wang F.. (2021b). Calcium-dependent protein kinase 32 gene maintains photosynthesis and tolerance of potato in response to salt stress. Scientia Hortic. 285, 110179. doi: 10.1016/j.scienta.2021.110179

