# Supplementary figures and images for: StMAPK1 functions as a thermos-tolerant gene in regulating heat stress tolerance in potato (Solanum tuberosum)
Source: Front Plant Sci. 2023 Jun 20;14:1218962. doi: 10.3389/fpls.2023.1218962 (PMC10319062; doi:10.3389/fpls.2023.1218962)

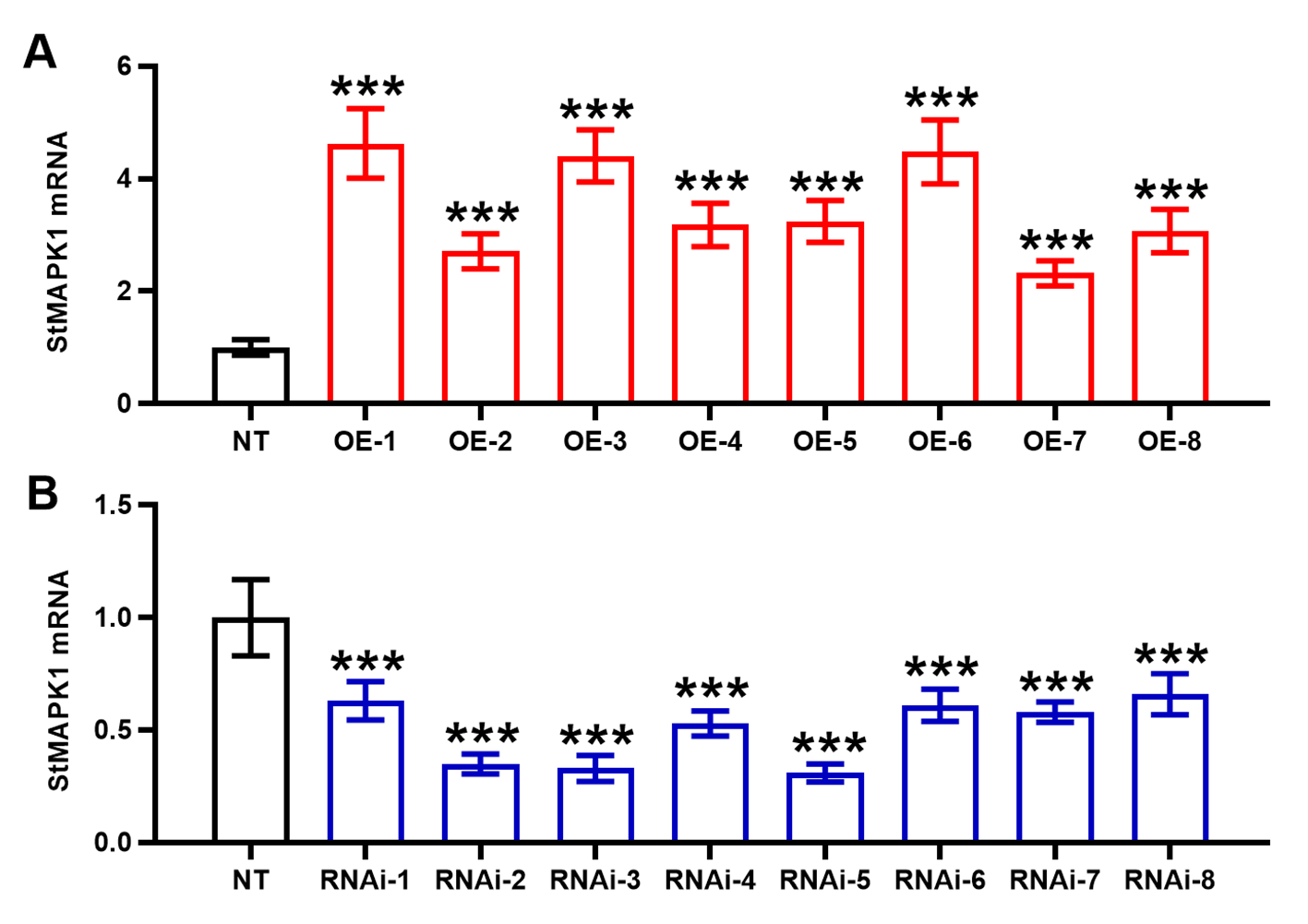

Supplement: Supplementary Figure 1 — mRNA expression of StMAPK1 in potato plants transfected with pBI121-EGFP plasmid carrying StMAPK1 protein-coding gene (A) and pART plasmid carrying anti-sense cDNA sequence of StMAPK1 gene (B). Data are mean ± standard deviation (n = 9). ***P < 0.001 (OE or RNAi compared to NC, two-way ANOVA corrected by Sidak’s multiple comparisons test). [file Image_1.tif]

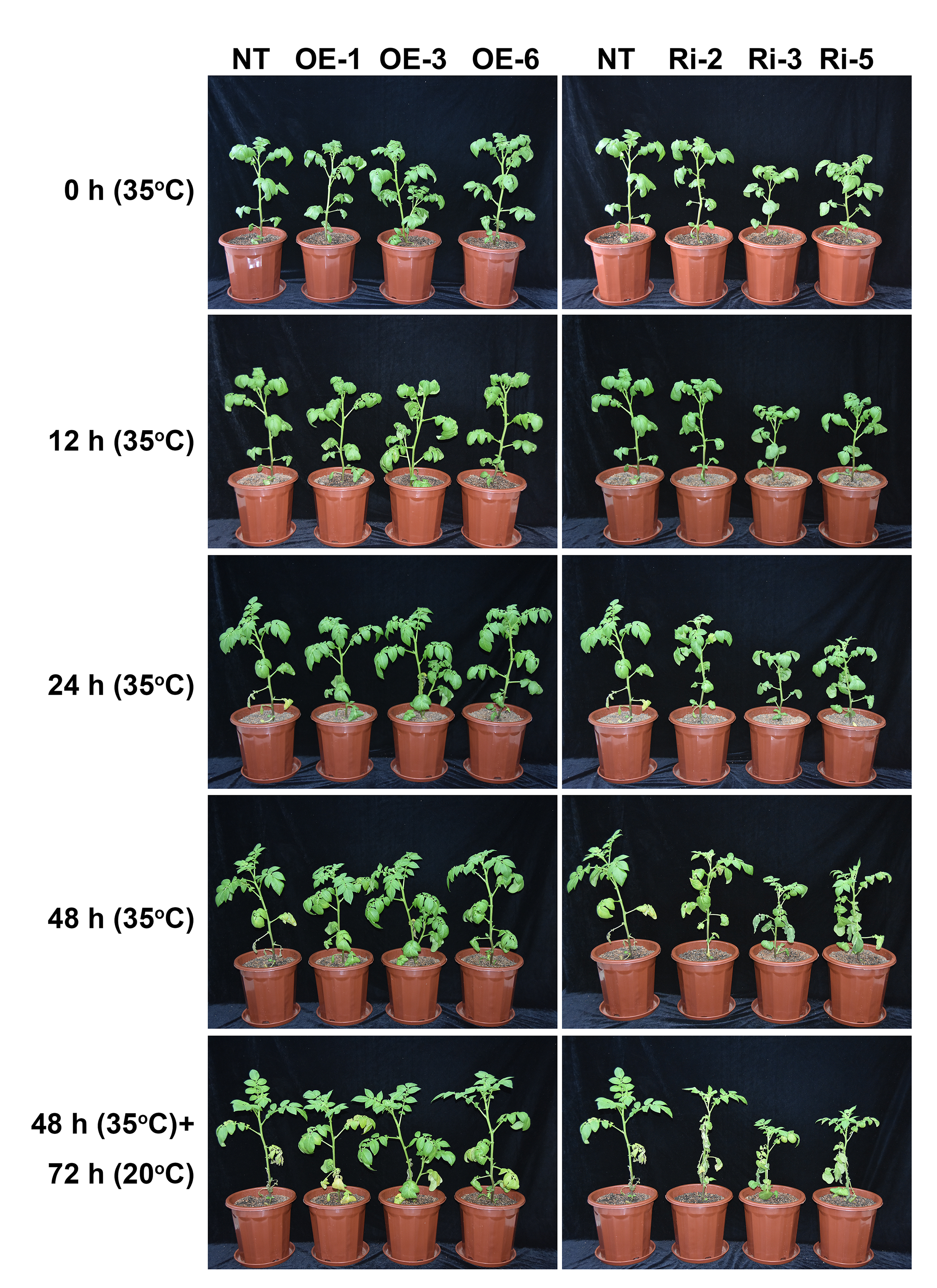

Supplement: Supplementary Figure 2 — Effects of StMAPK1 on potato phenotypes in response to heat stress. Phenotypic changes of potato plants cultivated 0 h, 12 h, 24 h, and 48 h after exposure to wild heat stress (35°C). The bottom group of potato pants was incubated at 35°C for 48 h, and then transferred to 20°C and cultivated for 72 h. Potato plants transfected with OE-1, OE-3 and OE-6 highly expressed StMAPK1 mRNA; Potato plants in Ri-2, Ri-3 and Ri-5 lowly expressed StMAPK1 mRNA. [file Image_2.tif]
