# Supplementary material for: StMAPK1 functions as a thermos-tolerant gene in regulating heat stress tolerance in potato (Solanum tuberosum)
Source: Front Plant Sci. 2023 Jun 20;14:1218962. doi: 10.3389/fpls.2023.1218962 (PMC10319062; doi:10.3389/fpls.2023.1218962)
Supplement: Supplementary file 3 [file Table_1.docx]

**Additional Table 1** Effects of *StMAPK1* on growth indexes of potato under heat stress

|  | Growth index | | | | | | | | | | | | | | |
| --- | --- | --- | --- | --- | --- | --- | --- | --- | --- | --- | --- | --- | --- | --- | --- |
|  | Plant height (cm) | | | Fresh weight (g) | | | Dry weight (g) | | | Root fresh weight (g) | | | Root dry weight (g) | | |
|  | 20 ^o^C | 30 ^o^C | 35 ^o^C | 20 ^o^C | 30 ^o^C | 35 ^o^C | 20 ^o^C | 30 ^o^C | 35 ^o^C | 20 ^o^C | 30 ^o^C | 35 ^o^C | 20 ^o^C | 30 ^o^C | 35 ^o^C |
| NT | 44.31±6.42b | 41.93±6.41b | 36.37±6.01ab | 183.08±26.63b | 163.15±22.27b | 148.17±19.89b | 38.42±5.48b | 33.27±4.67b | 30.29±4.78bc | 16.85±2.41b | 13.32±1.76b | 10.78±1.54b | 2.62±0.35b | 2.27±0.35b | 2.05±0.32b |
| OE-1 | 45.02±6.55b | 45.01±6.87b | 40.51±5.93b | 195.92±32.90b | 184.91±25.55b | 165.08±27.61b | 41.27±5.49bc | 39.21±6.18c | 34.83±5.16d | 18.46±2.77bc | 14.66±2.06b | 12.04±2.09b | 2.85±0.38b | 2.58±0.37c | 2.37±0.35c |
| OE-3 | 47.15±6.25b | 45.38±6.15b | 39.95±5.29b | 207.01±26.96b | 179.65±24.57b | 163.77±23.09b | 44.53±5.95c | 38.81±5.41c | 35.59±4.78d | 19.49±2.94c | 14.33±1.98b | 11.58±1.73b | 2.97±0.41b | 2.61±0.39c | 2.33±0.34bc |
| OE-6 | 46.26±6.38b | 44.89±5.9b | 39.76±5.34b | 200.61±26.01b | 184.16±25.52b | 160.12±21.21b | 43.32±5.61bc | 39.75±5.45c | 34.23±4.45cd | 18.33±2.38bc | 15.02±1.96b | 11.82±1.68b | 2.95±0.40b | 2.68±0.38c | 2.27±0.33bc |
| RNAi-2 | 37.14±4.93a | 35.44±4.63a | 31.95±4.78a | 140.39±18.62a | 125.98±18.3a | 118.82±16.8a | 31.07±4.26a | 27.47±3.56a | 25.24±4.03ab | 12.79±1.82a | 10.06±1.53a | 8.49±1.23a | 1.89±0.27a | 1.84±0.24a | 1.53±0.28a |
| RNAi-5 | 36.91±4.95a | 35.18±4.86a | 32.98±4.72a | 148.88±21.59a | 130.05±18.93a | 123.24±16.55a | 29.75±3.87a | 26.23±3.61a | 26.34±3.67ab | 13.58±2.28a | 10.29±1.40a | 8.89±1.22a | 1.92±0.25a | 1.74±0.25a | 1.51±0.22a |
| RNAi-6 | 36.63±5.01a | 34.64±4.87a | 31.51±4.62a | 138.29±18.97a | 129.84±19.65a | 120.53±19.96a | 30.88±5.10a | 26.10±3.40a | 26.03±4.00a | 13.17±1.98a | 10.33±1.57a | 9.03±1.27a | 2.01±0.31a | 1.81±0.26a | 1.66±0.22a |

Data are ± standard deviation (n = 9). Different lowercase letters represent the difference in the indicate growth index between two groups (P < 0.05, one-way ANOVA with Tukey test or Dunnett’s T3 for post-hoc analysis).
